# Dysmenorrhea among Nursing Staff in a Tertiary Care Center: A Descriptive Cross-sectional Study

**DOI:** 10.31729/jnma.6976

**Published:** 2021-08-31

**Authors:** Manoj Pokhrel, Meena Thapa

**Affiliations:** 1 Department of Obstetrics and Gynecology, Kathmandu Medical College and Teaching Hospital, Kathmandu, Nepal

**Keywords:** *dysmenorrhea*, *menstrual disorders*, *nursing*

## Abstract

**Introduction:**

Menstrual disorders are problems faced by women in their reproductive period, which affects their day-to-day activities and the quality of life and sometimes can be an indicator of serious pathology. There are various types of menstrual disorders with dysmenorrhea being the commonest and most distressing. Health, sense of wellbeing and quality of life can be improved because of early detection and treatment for these disorders. The study aimed to find out the prevalence of dysmenorrhea among the nursing staff of a tertiary care center.

**Methods:**

A descriptive cross-sectional study was carried out from September 2020 to November 2020 among the nursing staff of a tertiary care hospital. Ethical approval was taken from the Institutional review committee of Kathmandu Medical College and Teaching Hospital (reference number: 1709202003). A convenient sampling technique was used. A pre-structured questionnaire was used for data collection. The subjects were asked to fill the questionnaire anonymously after taking consent. Statistical analysis was done using the Statistical Package for the Social Sciences. Point estimate at 95% Confidence Interval was calculated along with frequency and percentage for binary data.

**Results:**

Out of 212 participants, 165 (77.8%) (95% Confidence Interval = 72.21-83.39) participants reported pain during menstruation. Of which, 61 (36.97%) participants reported abdominal pain.

**Conclusions:**

The prevalence of dysmenorrhea was high among the nursing staff of a tertiary care centre which was similar to the findings of other studies done in similar settings.

## INTRODUCTION

Menstruation is a process of cyclical shedding of the mucosal layer of the uterine endometrium associated with bleeding from the vagina which takes place during the reproductive age of a women.^1^ Menarche usually occurs between 11 to 14 years, the normal cycle length is about 21 to 35 days with a period length of 2-7 days or less and the average blood loss is around 20-80 ml.^2^ Menstrual disorders especially dysmenorrhea and heavy menstrual bleeding affect the quality of life of adolescents and young adults.^3^ These disorders have economic implications as there is an increase in health care costs due to expensive laboratory tests and the consumption of hormonal drugs.^4^

The findings of the study could help the nursing managers to provide a caring work environment for the nurses.

This study aims to find out the prevalence of dysmenorrhea among the nursing staff of a tertiary care centre.

## METHODS

A descriptive cross-sectional study was done among 212 nursing staff from September 2020 to November 2020 of Kathmandu Medical College and Teaching Hospital. Ethical approval was taken from the Institutional review committee of Kathmandu Medical College and Teaching Hospital (reference number: 1709202003). A convenient sampling technique was used. Nurses of the reproductive age group (15-45) of the Medical College working in different departments in Sinamangal and willing to participate in the study were included in the study.

Those participants with chronic health problems like endometriosis, bleeding disorders, psychiatric problems, any diagnosed pelvic pathologies like fibroids, pelvic inflammatory disease; pregnant and lactating mothers were excluded from the study.

Sample size calculation,

n = Z^2^ × p × q / e^2^

  = (1.96)^2^ × 0.5 × 0.5 / (0.07)^2^

  = 196

Where,

n = Sample size
Z = 1.96 at 95% Confidence Interval
p = prevalence of menstrual disorders for maximum sample size, 50%
q = 1 - p = 0.5
e = Margin of error, 7%

However, the total sample size taken was 212.

A pre-structured questionnaire was used for data collection. A structured, pre-designed questionnaire covering 24 items was used for the study. The questionnaire covered information about the following demographic variables: age, marital status and presence of pregnancy and breastfeeding to rule out the causes of amenorrhea. The subjects were asked to fill the questionnaire anonymously after taking consent. They were asked about their demographic profile, menstrual irregularities, pain during menstruation, site of pain, and remedies for pain. Statistical analysis was done using SPSS version 20. Point estimate is done at 95% Confidence Interval and frequency and proportion was calculated.

## RESULTS

Out of 212 participants, 165 (77.8%) (95% Confidence Interval = 72.21-83.39) participants reported pain during menstruation. Of which 61 (36.97%) participants reported abdominal pain (Table 1).

**Table 1:** Sites of pain during menstruation (n=165).

Characteristics	n (%)
Abdominal pain	61 (36.97)
Backache	36 (21.81)
Abdomen and back pain	60 (36.36)
Whole-body pain	4 (2.4)
Thigh pain	4 (2.4)

Table 2 shows the demographic profile of all the participants of our study. The majority of the participants 88 (41.5%) were from the age group 2630 years. Likewise, 138 (65.1%) participants were in normal weight limit with BMI in the range 18.5-24.9kg/m^2^ and 135 (63.7%) were married (Table 2).

**Table 2:** Demographic profile of participants (n=212).

Characteristics	n (%)
Age	
21-25yrs	52 (24.5)
26-30yrs	88 (41.5)
31-35yrs	36 (17.0)
36-40yrs	32 (15.1)
>40yrs	4 (1.9)
Total	212 (100.0)
BMI (Kg/m2)	
<18.5 (underweight)	6 (2.8)
18.5-24.9 (normal weight)	138 (65.1)
25-29.9 (over weight)	45 (21.2)
≥ 30 (obese)	23 (10.8)
Marital status	
Married	135 (63.7)
Unmarried	77 (36.3)

Among 212 participants, 161 (75.9%) reported stress due to night shifts and 186 (87.7%) reported stress due to work (Table 3).

**Table 3:** Stress due to night shift and work (n=212).

Characteristics	n (%)
Stress due to Night shifts	
Yes	161 (75.9)
No	51 (24.1)
Work stress	
Yes	186 (87.7)
No	26 (12.3)

Among 212 participants, 119 (56.1%) never took leave from the job. Likewise, 85 (40.1%) sometimes took leave from the job (Table 4).

**Table 4:** Leave from job (n = 212).

Leave from job	n (%)
Usually	8 (3.8)
Sometimes	85 (40.1)
Never	119 (56.1)

## DISCUSSION

The prevalence of dysmenorrhea or pain during menstruation is high in the study setting. Most of the participants complained of stress due to night shifts and work-related stress but 56.1% never took leave from job.

The prevalence of dysmenorrhea is high, 77.8%, of nursing staffs of our medical college which follows the study done by Hu Z, et al,^5^ Hashim RT, et al,^6^ and Chiu M.^7^ Nearly 40% of participants in our study took leave from job or college which is similar to the systematic review done by Armour M, et al.^8^

Most of the participants of our study, 75.9% of 212, reported stress due to night shifts and 87.7% of 212 reported stress due to work which follows the study done by Lawson CC, et al.^9^ which concludes that night shifts, long working hours, and physically demanding work may be the cause to menstrual disturbances among the nursing staffs. Adverse health symptoms of menstrual disorder represent significant occupational health challenges in the nursing profession.^10^

This is a study done among nursing staff of a single hospital, so, the result might not be generalized to all the health care personnel, for which multi-center study among the nursing staff is necessary. There may be recall bias by participants due to which the data may not be accurate.

## CONCLUSIONS

The prevalence of dysmenorrhea is high among the nursing staff of a tertiary care center which is similar to the findings of other studies done in similar settings. The findings of this study may be helpful for the nursing managers to provide a caring work environment for the nurses. Since nursing profession includes females, so, menstrual disorders and their adverse symptoms represent a significant occupational health challenge in the profession.
